# Role of NOX2-derived ROS in the development of cognitive impairment after sepsis

**DOI:** 10.1186/cc12996

**Published:** 2013-11-05

**Authors:** Joana C D'Avila, Marina S Hernandes, Silvia C Trevelin, Patricia A Reis, Hugo Castro-Faria-Neto, Fernando Q Cunha, Luiz RG Britto, Fernando A Bozza

**Affiliations:** 1 Oswaldo Cruz Institute, FIOCRUZ, Rio de Janeiro, Brazil; 2 Department of Pharmacology, Institute of Biomedical Sciences, University of São Paulo, Brazil; 3 Department of Pharmacology, Faculty of Medicine of Ribeirão Preto, University of São Paulo, Ribeirão Preto, SP, Brazil; 4 Evandro Chagas Clinical Research Institute (IPEC), FIOCRUZ and D'Or Institute for Research and Education (IDOR), Rio de Janeiro, Brazil

## Background

Septic encephalopathy (SE) is a frequent complication in severe sepsis. Here we have explored the role of NADPH oxidase in different aspects of SE pathophysiology. We investigated the involvement of NADPH oxidase in neuroinflammation and in the long-term cognitive impairment of sepsis survivors.

## Materials and methods

Our approach included pharmacological inhibition of NADPH oxidase activity with apocynin and the use of genetically deficient (knockout) mice for gp91phox (gp91phox^-/-^), the catalytic subunit of Nox2. Sepsis was induced by cecal ligation and puncture and fecal peritonitis. We measured the hippocampal oxidative stress, Nox2 and Nox4 gene expression and neuroinflammation in WT and gp91phox^-/- ^mice at 6 hours, 24 hours and 5 days post sepsis. Behavioral outcomes were evaluated 15 days after sepsis with the inhibitory avoidance and the Morris water maze tests.

## Results

The data show progressive oxidative damage to the hippocampus, identified by increased 4-hydroxynonenal expression, associated with an increase in Nox2 gene expression in the first days after sepsis. Pharmacological inhibition of Nox2 with apocynin completely inhibits hippocampal oxidative damage in septic animals as well as the development of long-term cognitive impairment in the survivors. Pharmacologic inhibition or the absence of Nox2 in gp91phox^-/- ^mice prevents glial cells activation, one of the central mechanisms associated with SE and other neurodegenerative diseases.

## Conclusions

We identified Nox2 activation as a necessary step for glial cell activation in SE. Our data indicate that Nox2 is as a major source of oxidative stress in the brain and consequently has a central role in the development of cognitive impairments observed in sepsis survivors.

